# Supplementary material for: SNARE Protein DdVam7 of the Nematode-Trapping Fungus Drechslerella dactyloides Regulates Vegetative Growth, Conidiation, and the Predatory Process via Vacuole Assembly
Source: Microbiol Spectr. 2022 Oct 26;10(6):e01872-22. doi: 10.1128/spectrum.01872-22 (PMC9769606; doi:10.1128/spectrum.01872-22)
Supplement: Supplemental file 6 — Fig. S1-S7; Tables S1 and S2. Download spectrum.01872-22-s0006.pdf, PDF file, 1.4 MB [file spectrum.01872-22-s0006.pdf]

Supplementary Information

Fig. S1

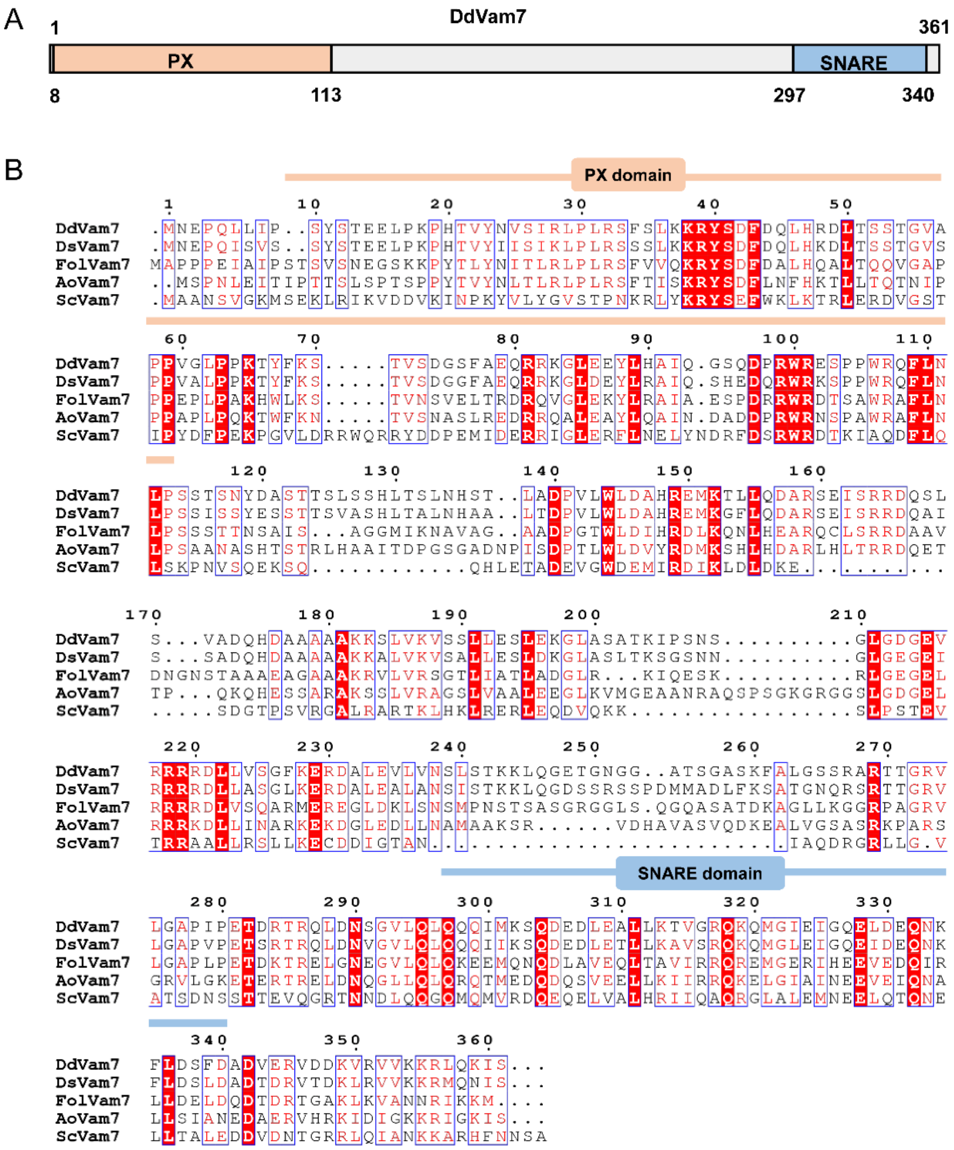

Fig. S1 Sequence alignment of *D. dactyloides* DdVam7 with its homologues encoded by other species. (A) A schematic diagram of DdVam7 and its PX and SNARE domains. The PHOX homology motif (PX) (8–113 residues) at the N-terminus and a SNARE domain (297–340 residues) at the C-terminus are noted. (B) Sequences of DdVam7, DsVam7 (*Drechslerella stenobrocha*, EWC44037.1), FolVam7 (*Fusarium oxysporum* f. sp. *lycopersici*, XP_018235815.1), AoVam7 (*Aspergillus oryzae*, BAF36378.1), and ScVam7 (*Saccharomyces cerevisiae*, NP_011303.1) were aligned using ClustalW. Conserved amino acids are in blue boxes. Similar amino acids are indicated by red text, and identical amino acids are noted using a red background.

Fig. S2

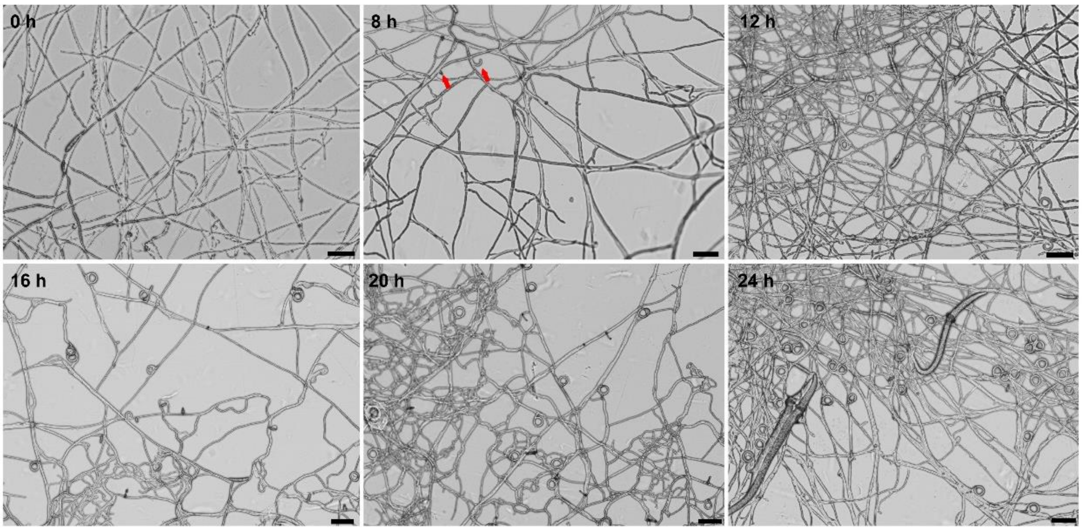

Fig. S2 CR formation after introducing *C. elegans* on *D. dactyloides* mycelia growing on WA plates covered with cellophane membrane. Images were collected at 0, 8, 12, 16, 20 and 24 hours after introducing *C. elegans*. Red arrows indicate incompletely formed CRs. Scale bar = 50 μm.

Fig. S3

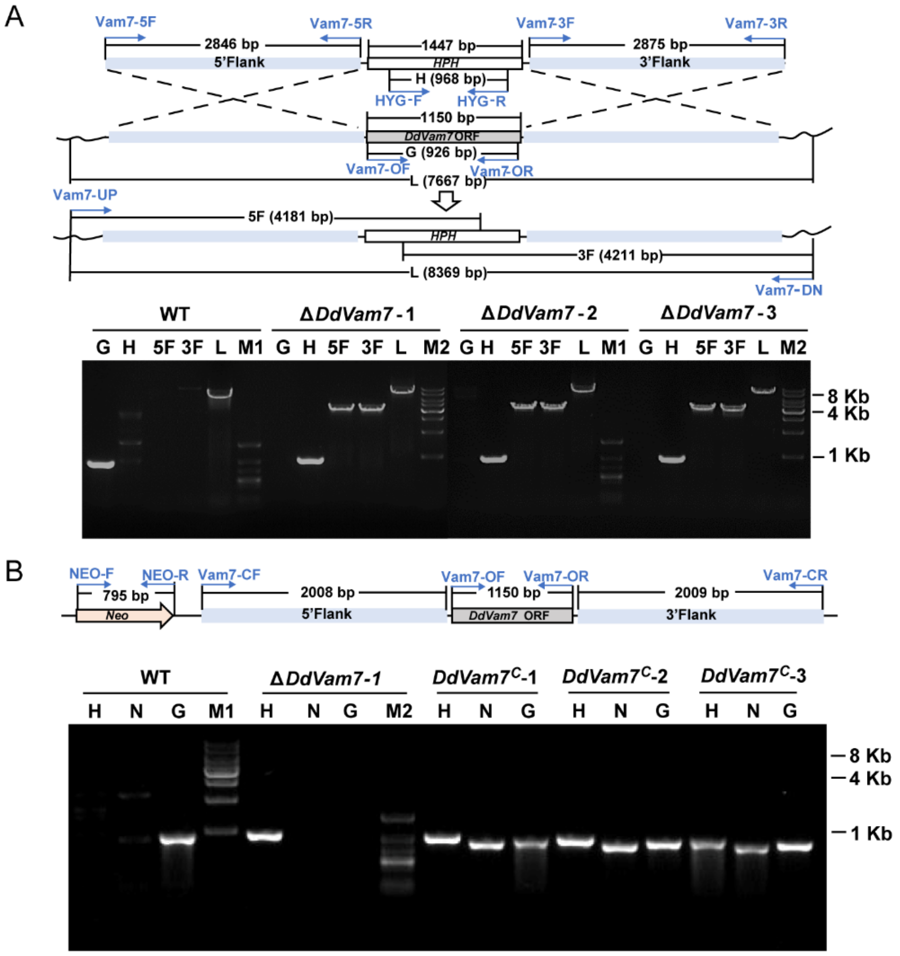

Fig. S3 Disruption and complementation of *DdVam7*. (A) Disruption of *DdVam7* via homologous recombination (upper panel) and verification of gene disruption using PCR (lower panel). (B) Complementation of Δ*DdVam7* (upper panel) and verification of complementation using PCR (lower panel). M1: DNA marker D2000; M2: DNA marker 1 Kb; H: partial hygromycin resistance gene amplified using primers HYG-F and HYG-R; N: partial geneticin resistance gene amplified using primers NEO-F and NEO-R; G: partial *DdVam7* ORF amplified using primers Vam7-OF and Vam7-OR; 5F: Correct recombination ensured by PCR using primers Vam7-UP and HYG-R; 3F: Correct recombination ensured by PCR using primers Vam7-DN and HYG-F; L: PCR results amplified using primers Vam7-UP and Vam7-DN. The regions amplified via PCR and the locations of individual primers were marked in upper panel.

Fig. S4

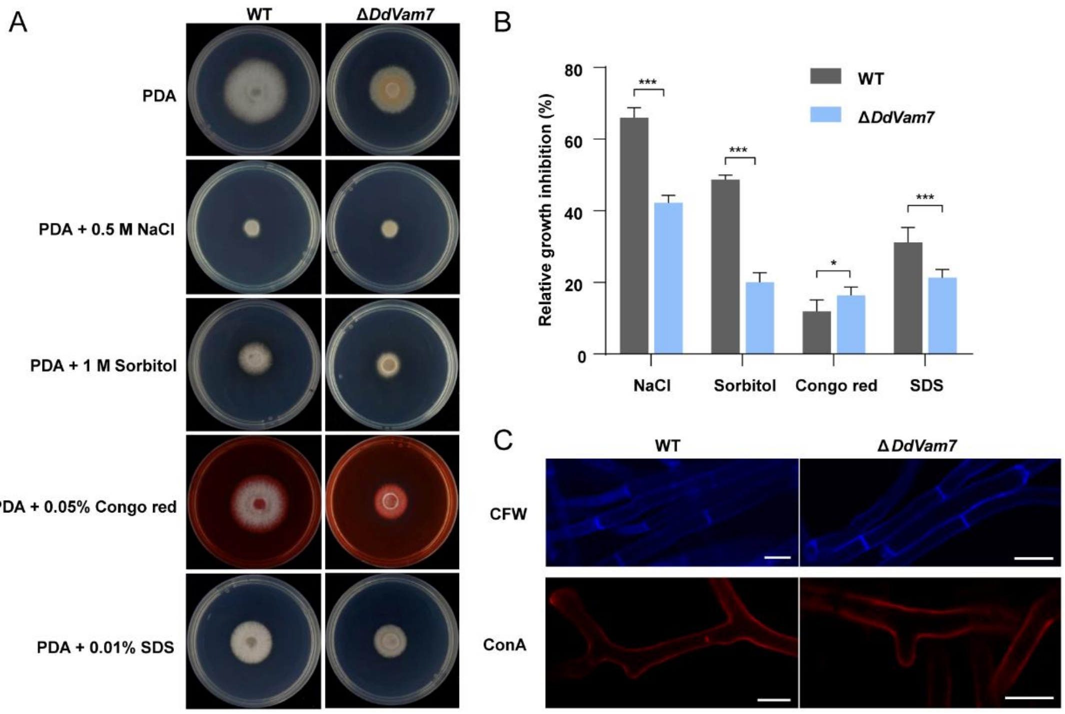

Fig. S4 Role of DdVam7 in responding to abiotic stress. (A) Colony morphology of the wild type (WT) and Δ*DdVam7* inoculated on PDA medium plates alone or supplemented with stressors. (B) Bar chart showing the relative growth inhibition by individual stressor. ***$P < 0.001$, *$P < 0.05$ ($n = 6$). (C) Micrographs showing hyphae of the WT and Δ*DdVam7* strains stained with CFW (for detecting chitin) and ConA (for staining α-mannopyranosyl and α-glucopyranosyl residues). The data shown here are representative results from the experiments performed in duplicate. Scale bar = 5 μm.

Fig. S5

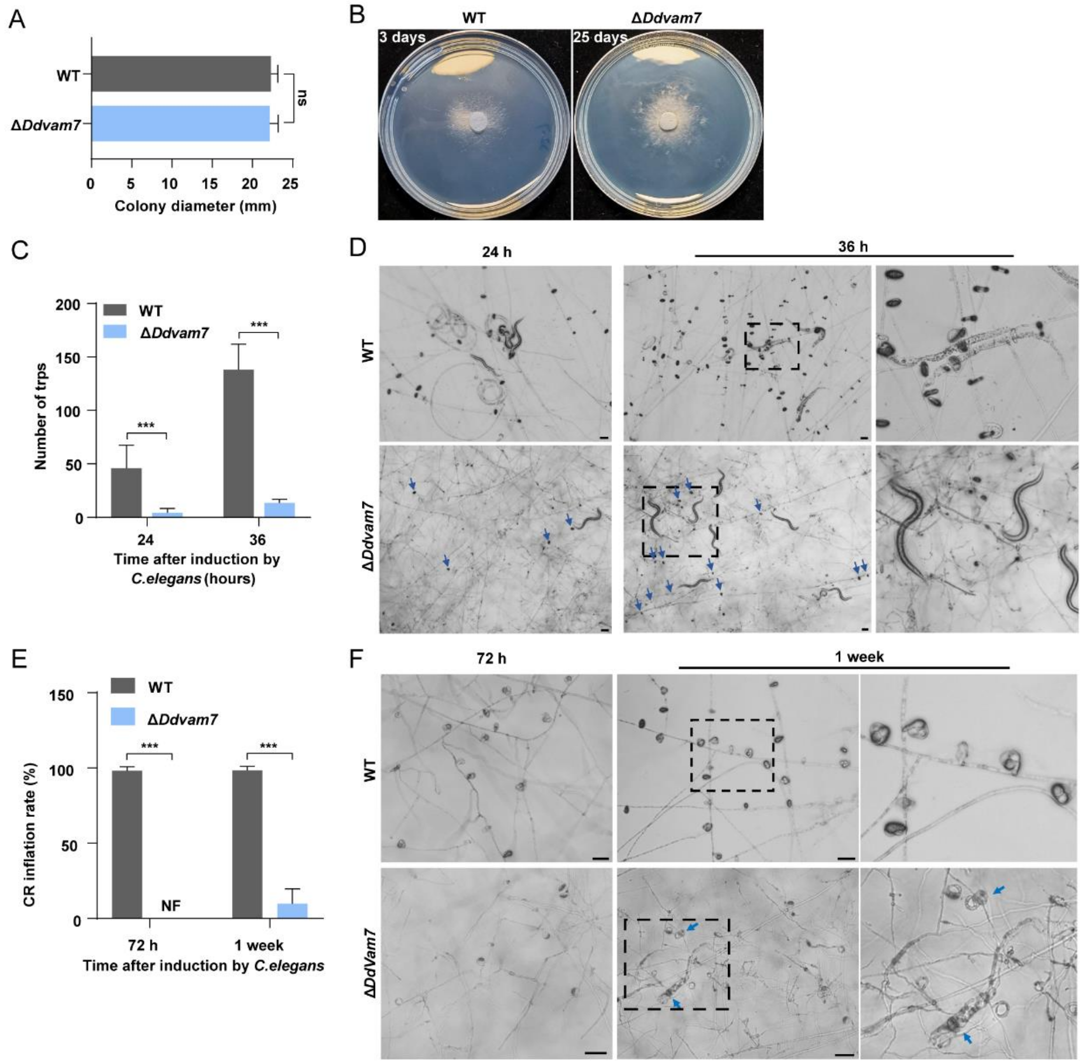

Fig. S5 Phenotypic comparison between Δ*DdVam7* and wild type under comparable growth. (A) Colony diameters of wild type (WT, 3-days-old culture) and Δ*DdVam7* (25-day-old culture). ns = not significant (n = 5). (B) Colony morphology of WT and Δ*DdVam7* cultured on WA plates for 3 days and 25 days respectively. (C) The numbers of CRs formed at 24 and 36 hours after introducing *C. elegans*. ***P < 0.001 (n = 6). (D) Light micrographs showing CR formation capacity. Blue arrows indicate the CRs. The right panel is close-up view indicated by dotted black box. Shown are representatives of at least five images. Scale bars = 50 µm. (E) The percentages of inflated CRs at 72 hours and 1 week after introducing *C. elegans*. ***P < 0.001, NF = not found (n = 6). (F) Light micrographs showing ring cell inflation. Red arrows indicate inflated CRs. Scale bars = 50 µm.

Fig. S6

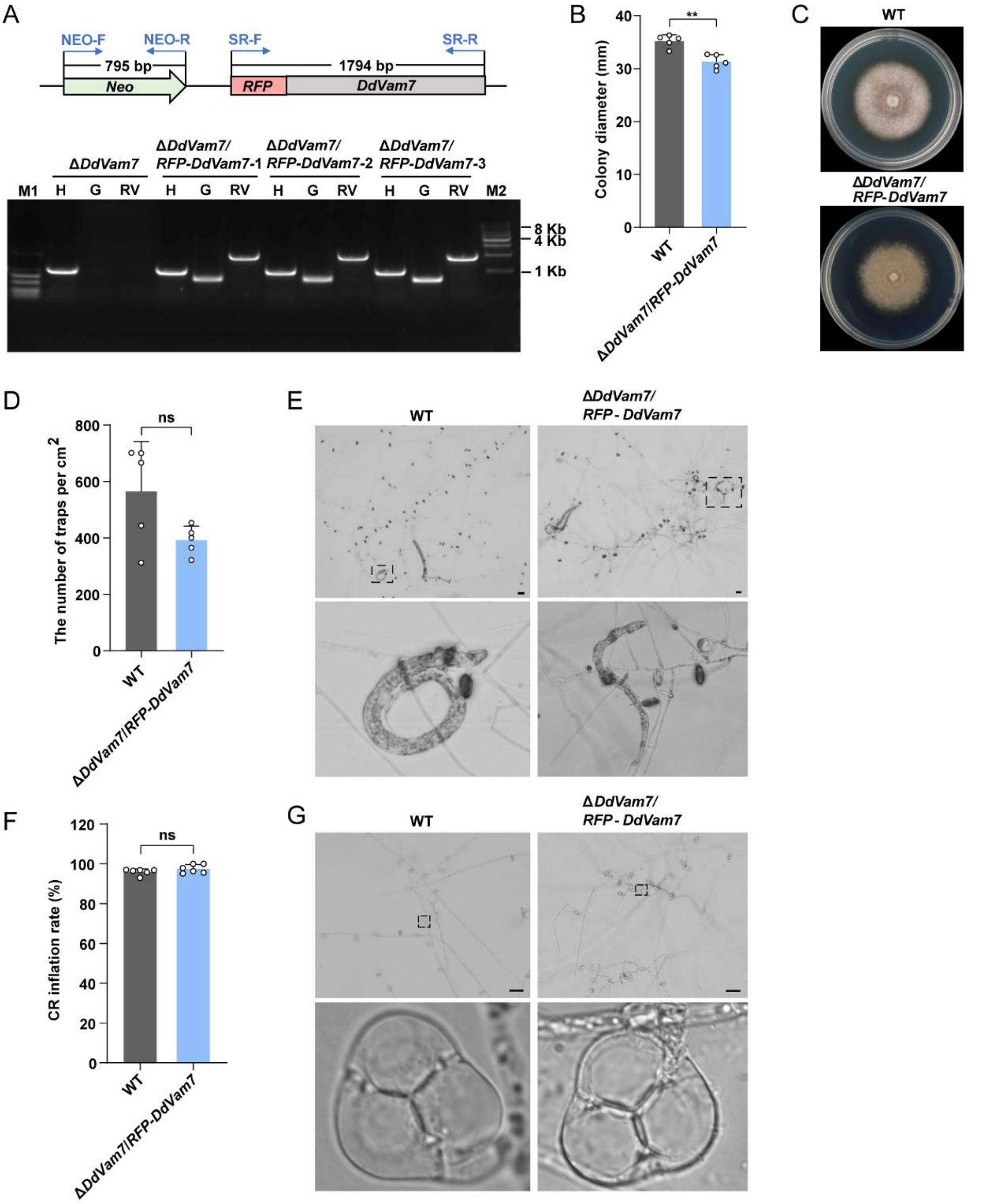

Fig. S6 Phenotypic characteristics of the Δ*DdVam7*/*RFP-DdVam7*. (A) Construction of Δ*DdVam7*/*RFP-DdVam7* strains. M1: DNA marker D2000; M2: DNA marker 1 Kb; H: partial hygromycin resistance gene amplified using primers HYG-F and HYG-R; G: partial geneticin resistance gene amplified using primers NEO-F and NEO-R; RV: PCR results amplified using primers SR-F and SR-R. The regions amplified via PCR and the locations of individual primers were marked in upper panel. (B) Colony diameters of wild type (WT) and Δ*DdVam7*/*RFP-DdVam7* cultured on PDA plates for 10 days. **P < 0.01 (n = 5) (C) Colony morphology of WT and Δ*DdVam7*/*RFP-DdVam7* cultured on PDA plates for 10 days. (D) The numbers of CRs formed at 36 hours after introducing

C. elegans. ns = not significant (n = 5). (E) Light micrographs showing CR formation capacity at 36 hours after introducing *C. elegans*. The lower panel is close-up view indicated by dotted black box. Shown are representatives of at least five images. Scale bars = 50 μm. (F) The percentages of inflated CRs at 72 hours after introducing *C. elegans*. ns = not significant (n = 6). (G) Light micrographs showing ring cell inflation. The lower panel is close-up view indicated by dotted black box. Scale bars = 50 μm.

Fig. S7

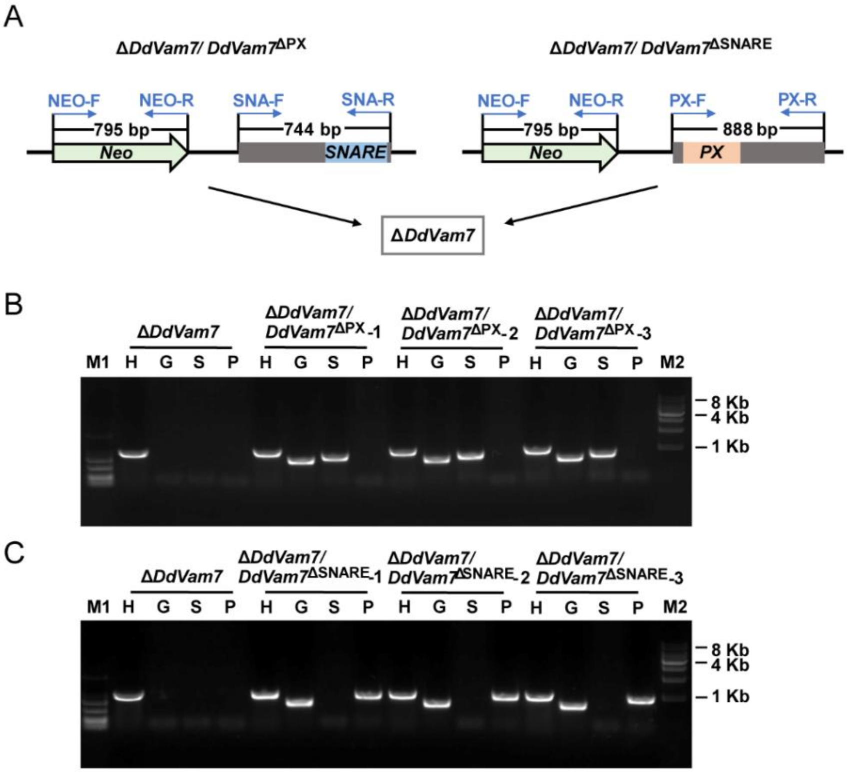

Fig. S7 Domain deletion. (A) Schematic diagram of the domain deletion strategy. (B, C) Verification of domain disruption using PCR. M1: DNA marker D2000; M2: DNA marker 1 Kb; H: partial hygromycin resistance gene amplified using primers HYG-F and HYG-R; G: partial geneticin resistance gene amplified using primers NEO-F and NEO-R; S: PCR results amplified using primers SNA-F and SNA-R; P: PCR results amplified using primers PX-F and PX-R. The regions amplified via PCR and the locations of individual primers were marked in (A).

Table S1. List of putative SNARE proteins in *D. dactyloides*.

Locus name	Annotated function	Length (a.a)	SNARE domain position
1295_g_dac	Syntaxin/t-SNARE Sso1	309	208-271
2995_g_dac	Syntaxin/t-SNARE Sso1	470	371-433
3420_g_dac	Synaptobrevin/Vesicle-trafficking protein Sec22	215	133-212
3592_g_dac	Synaptobrevin/VAMP-like protein/v-SNARE SNC1	237	141-227
3734_g_dac	Qc-SNARE Vam7	361	297-340
4406_g_dac	Golgi SNAP receptor complex member 2 (GOSR2)	329	241-302
5041_g_dac	t-SNARE Syntaxin5 (Syn5)	321	266-317
5412_g_dac	v-SNARE VTI1 family protein	226	21-98
5465_g_dac	t-SNARE Syntaxin-2	272	199-239
621_g_dac	t-SNARE Syntaxin-16	419	236-296
65_g_dac	t-SNARE Syntaxin-8	306	219-286
7439_g_dac	SNARE Syntaxin-18	352	265-323
7538_g_dac	Synaptobrevin/VAMP-like protein/v-SNARE SNC1	119	29-88
8115_g_dac	t-SNARE Syntaxin-6	240	147-208
8656_g_dac	Golgi SNAP receptor complex member 1 (GOSR1)	225	141-202
8744_g_dac	Synaptobrevin/R-SNARE YKT6	207	145-205

Table S2. List of the primers used in this study.

Primers	Sequence (5'-3')	Description
Vam7-5F	GTAACGCCAGGGTTTTCCCAGTCACGATC GGATAGCAGTCAGCAGTGGAT	Amplify the 5’ flanking region of the *DdVam7* gene
Vam7-5R	CGAATTCACTGGCCGTCGTTTTACAACGT CCGAGTAGTAAGTCCTGGATGTC	
Vam7-3F	CGAAAATCATTCCTACTAAGATGGGTATA CGCATATACGCACGCGCAATTTTC	Amplify the 3’ flanking region of the *DdVam7* gene
Vam7-3R	CGGAGCATTCACTAGGCAACCATGGTTAC TATTGGTTGACGCACATGTCCCATG	
Vam7-CF	AGATCTAGAGGATCCCCCGACTAGTAGCC ACATCGAGAGGTCTTGTTGTG	Amplify the DdVam7 coding region
Vam7-CR	ATTTAAATCCGTTTAAACGGCGCGCCATG AAGTCGCCAGCGATTACTGTGC	
Vam7-OF	ACCGTCTACAACGTCAGCATCCGG	Amplify the *DdVam7* ORF
Vam7-OR	AGTTCCTGCCCTATCTCAATGCCC	
HYG-F	AACTCACCGCGACGTCTGTCGAG	Amplify the hygromycin resistance gene
HYG-R	ATCGGCGAGTACTTCTACACAGCCAT	
NEO-F	GGCTATGACTGGGCACAACA	Amplify the geneticin resistance gene
NEO-R	GATACCGTAAAGCACGAGGAA	
Vam7-UP	AGTCGTCGCTGTAGCAGCCATATTGG	Verify the transformants (Amplify the 5F, 3F and L)
Vam7-DN	TCGCTGGCACCGATGTTTGGTAGTC	
Vam7-Q-F	ACCGTCTACAACGTCAGCATCCGG	For RT-PCR analysis of *DdVam7*
Vam7-Q-R	AGGAGCCACACCAGTCGACGATG	
SNA-F	TCGTCTACATCCAATTACGACGCTTC	Amplify the DdVam7$^{\Delta PX}$ coding region
SNA-R	TCAGCTGATTTTTTGTAGGCGTTTCTTGACC	
PX-F	ATGAACGAGCCACAGCTCTTGA	Amplify the DdVam7$^{\Delta SNARE}$ coding region
PX-R	TCACAGCTGTAAGACGCCCGAATTAT	
SR-F	GAGCAAGGGCGAGGAGGATA	Amplify the RFP-DdVam7 coding region
SR-R	TATCGTCAACCCGTTCAACGTC	

Supplementary Movie Legends

Movie S1. A nematode getting caught by a CR in wild type.

Movie S2. A nematode getting caught by a CR in *DdVam7*^{C}.

Movie S3. A nematode entering and escaping from a CR of Δ*DdVam7*.

Movie S4. Complete CR inflation in *D. dactyloides*, related to Figure 4D.

Movie S5. Uncomplete CR inflation in *D. dactyloides*, related to Figure 4E.
